# The response of inclusive schools to the needs of children exposed to Adverse Childhood Experiences

**DOI:** 10.3389/fpsyg.2025.1671967

**Published:** 2025-11-07

**Authors:** Irune Corres-Medrano, Klara Smith-Etxeberria, Itziar Fernández-Villanueva

**Affiliations:** 1 Department of Basic Psychological Processes and Their Development, University of the Basque Country (UPV/EHU), Donostia-San Sebastián, Spain; 2 Department of Social Psychology, University of the Basque Country (UPV/EHU), Donostia-San Sebastián, Spain

**Keywords:** Adverse Childhood Experiences (ACE), children at risk, emotional instability, school segregation, socio-emotional development, inclusive school

## Abstract

This research seeks to explore the bidirectional adaptation of the educational community in schools that take in children at risk and who have suffered ACE. On the one hand, it describes the psychological adjustment of children at risk to the school context. On the other hand, it reports on the school's response to the needs of these children. Three public primary schools characterized by social and school segregation participated in this study. Participants included 25 primary education professionals and 144 third- and fourth-year primary school children, from diverse cultural backgrounds. Data collection techniques consisted of participant observation, semi-structured interviews and focus groups. The analysis revealed the following five themes: (1) emotional instability of students; (2) the crisis of authority questions the place of the teacher; (3) difficulties in good teaching practices; (4) grouping and segregation of students; and (5) need for other material and human resources on the part of teachers. Findings suggest the need to develop comprehensive and systemic lines of research and intervention that take into account both the individual characteristics of children and the social factors that characterize schools. In addition, the fundamental role played by inclusive schools in addressing the rights of these most vulnerable children and ensuring their well-being is highlighted.

## 1 Introduction

Children need optimal biological and social conditions that guarantee their wellbeing (Martorell et al., 2013; Narvaez, 2018). In all childhood descriptions, a series of basic developmental needs are recognized, among which those referring to biological care and socio-emotional needs are highlighted (Blodgett and Lanigan, 2018; López, 2008). Hence, it is vitally important to facilitate and favor the child's development process in the best conditions (Williams, 2017).

However, some children are deprived of optimal development that meets all their needs, due to Adverse Childhood Experiences (ACE). These experiences include abuse (sexual, physical or emotional abuse), neglect (physical or emotional), abandonment, mental or addiction problems by primary caregivers, gender-based violence, and troubled divorces, among others (Felitti et al., 1998; van der Kolk et al., 2009).

Unmet basic emotional, social and cognitive needs derived from ACE may negatively affect brain development, cognitive development, learning, socio-emotional development, the ability to develop secure attachments with others and/or physical health (Garay et al., 2022; Rahapsari and Levita, 2024; Webster, 2022). Indeed, the earlier the neglect and exposure to trauma begins, the more damaging and severe the effects can be, and the deeper and more difficult the recovery will be (Arruabarrena et al., 2019; Dym and Steber, 2019). Likewise, scientific literature shows that the accumulation of ACE generates toxic stress (Do Prado et al., 2017), which increases the risk of developing health problems in various areas, including emotional, physical, neurological, and behavioral (Atkinson et al., 2015; Felitti et al., 1998; Hughes et al., 2017). Therefore, children must receive protection and care at an early age. Otherwise, they remain at risk, which exposes them to a greater risk of exhibiting disruptive behaviors (e.g., violence, sexual abuse, and drug use) or internalizing problems in the near future (Diamond, 2013; Dym and Steber, 2019; Gooch et al., 2016; Pereda et al., 2014).

### 1.1 Impact of ACEs on learning

The international literature consistently highlights that children at risk are at heightened risk of educational underachievement, due to the impact of trauma on cognitive, emotional, and behavioral functioning (Ismail, 2019; Roseby and Gascoigne, 2021). Furthermore, students with adverse experiences, who attend schools characterized by segregation and social vulnerability show the following behavioral and emotional manifestations: lack of autonomy, initiative and motivation; constant calls for attention or need for support for simple tasks; difficulties with attention and concentration; poor and unstable habits of order, work and discipline; along with hyperactivity and difficulties in social relations with peers (Buchanan-Pascall et al., 2018; Mundy et al., 2017; Wade et al., 2022).

In spite of these results, there is ample evidence on people's capacity to adapt to early adversity, particularly when protective factors such as stable relationships, supportive environments, and opportunities for skill-building are present (Bartlett et al., 2021; Masten, 2014; Masten et al., 2023). Indeed, current research suggests that most children at risk do not experience drastic outcomes, as many exhibit resilient factors that protect them from negative consequences (Giovanelli et al., 2020; Masten and Labella, 2016; Russell et al., 2020). However, despite these children's intrinsic capacity, given their vulnerability, children must be protected by various support systems (Dolan et al., 2020; Harvey et al., 2021). As Garay et al. (2024) emphasize, trauma-informed schools must act as protective environments that integrate educational, emotional, and social support to prevent re-traumatization and foster resilience in children exposed to adversity.

### 1.2 The role of schools as protective environments

Among the various systems of support for children, alongside family, schools also stand out as one of the main agents of socialization. Nevertheless, in order for the schools to occupy this place of protection it must meet certain conditions, such as promoting the fundamental human universal right of inclusion (UNESCO, 1990). That is, schools are an exceptional context for the development and acquisition of social-emotional skills for overcoming adverse experiences (Merrigan and Senior, 2023).

Inclusive education stems from the Salamanca Statement on Special Needs Education, which emerged from the Education for All (EFA) movement (UNESCO, 1994) and aims to provide additional resources to help students with functional diversity, learning difficulties and socio-emotional disadvantages to access the curriculum and succeed in school (Powell, 2011). Recent research emphasizes that inclusive education must address not only access to education but also the emotional and social wellbeing of students, particularly those at disadvantage or at risk, through context-sensitive and equitable practices (Taneja-Johansson and Singal, 2025). However, governments do not always deliver on the right to education so not all schools exercise the right to inclusion to the same level (UNESCO, 2019). While some schools make significant contributions to the promotion of social justice, others contribute to the maintenance of social inequalities.

Despite the irrefutable international agreement that inclusive education is a right for all students (Amor et al., 2019), there are still real routes or pathways of exclusion, as shown by school segregation. The high concentration of migrant students in schools continues to reflect the residential criteria of a traditional catchment area model (Rözer and Van de Werfhorst, 2017; van der Werf et al., 2021). Thus, in neighborhoods characterized by being highly socioeconomically segregated, schools also tend to be highly segregated. These policies have the common goal of creating a certain degree of academic homogeneity within classrooms or schools (Nusche, 2009; Pàmies and Tarabini-Castellani, 2017). As a result, these schools group students in situations of inequality (e.g., migrants, students with disabilities, etc.) in separate environments isolated from students who do not have this type of disadvantage (UN, 2016).

### 1.3 Challenges of inclusive education

The challenges of today's society constantly place new demands on schools and their teachers, requiring them to become more socially involved. For example, given that students with ACE show emotional instability, difficulties with emotional regulation, attention problems, relationship difficulties and problems with executive functions (Lund et al., 2020; Yeo et al., 2024), they are often considered disruptive and difficult. Therefore, teachers might feel helpless in the face of student dysregulation, especially if they have not been specifically trained and sensitized to social vulnerability and child protection (Garay et al., 2022). In addition to the difficulties faced by teachers, the school's functioning and organization, alongside staff instability, excessive workload due to the demands resulting from the diversity of children's needs, lack of time and lack of family involvement might also be an additional difficulty. Teachers therefore respond to the diverse needs of the educational community with their own skills and the resources at their disposal (Downes, 2011). As a result, all of this can affect teachers' involvement, motivation and commitment to their work with children and to the school's projects (Baker et al., 2016; Jellison et al., 2017).

## 2 Current study

Despite extensive research on ACE and their impact on child development, to our knowledge, limited attention has been given to how these experiences affect educational dynamics from teachers' perspective, especially in inclusive school contexts. Specifically, very few studies have focused on examining the processes of two-way adaptation between at-risk students and educational institutions, understood as the mutual adjustment between children's individual needs and schools' structural and pedagogical responses (Castilla Mesa et al., 2022). Hence, this study attempts to contribute to the literature by overcoming these caveats and focusing on the Spanish context, where phenomena such as school segregation and cultural diversity pose specific challenges (Murillo and Belavi, 2021). By analyzing the protective and vulnerability factors that impact the teaching-learning process and the socio-emotional development of at-risk children, this research offers a comprehensive perspective that contributes to the knowledge needed to guide more inclusive educational policies that are sensitive to contemporary social realities.

Thus, the main aim of this study is to analyze protective and vulnerability factors that condition the teaching-learning process and the socio-emotional development of children at risk who attend primary schools. To this end, first, the study analyzes the response of inclusive schools to the needs of these students. In addition, it analyzes the impact of social factors, such as school segregation (i.e., isolation from the native population) on their socio-emotional development.

## 3 Methodology

### 3.1 Participants

Participants in the current study included three segregated schools in the city of Vitoria-Gasteiz (Basque Autonomous Community or Basque Country, Spain). In recent years, real poverty has increased considerably in this city (Social Services Observatory of Alava, 2021). Real poverty includes those circumstances in which the situations of risk of insufficient coverage of basic needs that appear in one or other of the different dimensions of poverty (maintenance or accumulation) are not sufficiently compensated in the daily life of the population in such a way that it is possible to access a minimum level of wellbeing, outside the experience of poverty (Basque Government, 2024). The three participating segregated schools were selected using purposive sampling, given that the study is focused on highly vulnerable school contexts that are representative of educational segregation in Vitoria-Gasteiz. That is, these schools were chosen because they had a particularly low Socio-Economic and Cultural Index (ISEC), as well as a high concentration of students in risk of poverty and/or exclusion (AROPE) situation. The ISEC is an indicator of the socio-economic and cultural level of the families. In the case of Vitoria-Gasteiz, although from 2011 to 2019, there has been a slight decrease, during this same period, the average ISEC of private schools has increased (Irurrebaso et al., 2023). A person or a family is in an AROPE situation (at risk of poverty and/or exclusion) if he/she meets at least one of the following three criteria: at risk of poverty, severely materially and socially deprived, or aged 0–64 and living in a household with low employment intensity [European Anti Poverty Network, Spain (EAPN-ES), 2023]. Therefore, although the number of schools in this research is limited, their inclusion responds to criteria of contextual relevance, which allows an in-depth exploration of the social and educational dynamics affecting at-risk children. Furthermore, these schools reflect structural patterns present in other urban areas with similar characteristics, such as the polarization between public and private schools, the impact of real poverty on the school environment, and the ethnic and cultural diversity of the student body. Therefore, although the study does not aim to generalize its results to the entire segregated school population, it does offer a significant sample of critical cases, which allows for a deeper understanding of the challenges and opportunities of inclusive schools in contexts of high social vulnerability.

This study included 144 third- and fourth-year primary school children from three schools, which represented a wide variety of ethnic and cultural backgrounds. In addition, 25 people from different professional backgrounds within the educational community such as directors, support teachers, and school counselors participated (*M* age =42.44 years; *SD* = 11.57). Within the participating education professionals, 18 identified themselves as women and 7 as men. Regarding their professional experience, these participants reported having had an average of 13.84 years of experience (*SD* = 11.11).

### 3.2 Research methods and information collection procedures

This investigation was based on a qualitative methodology, as it allows to analyze the phenomenon of interest in depth (Flick, 2014). In particular, we conducted an ethnographic research, which is characterized by the naturalness and flexibility of the research process through which data collection techniques are selected and constructed (Haven and Van Grootel, 2019; LeCompte and Goetz, 1993). From that flexibility, in this study we chose to collect information through participant observation (Denzin and Lincoln, 2017; LeCompte and Goetz, 1993), interviews (Flick, 2014) and focus groups (Flick, 2018).

Hereunder, Table 1 shows the tools used, the contexts analyzed and the profile of the people involved in the data collection.

**Table 1:** Data collection, research participants, and features.

**Tools**	**Profile and characteristics of participants^a^**	***n***	
Participant observation	Students	144 students	**A school:** Group 3rd: 25 (*n* = 12 boys; *n* = 13 girls) Group 4th: 23 (*n* = 11 boys; *n* = 12 girls) Ethnic groups: Morocco, Sahara, Nigeria, Cameroon, Bolivia, China, Spain
			**B school:** Group 3rd: 25 (*n* = 12 boys; *n* =13 girls) Group 4th: 24 (*n* =12 boys; *n* =12 girls) Ethnic groups: Morocco, Nigeria, Cameroon, Bolivia, China, Spain, Romas
			**C school:** Group 3rd: 25 (*n* = 13 boys; *n* = 12 girls) Group 4^th^: 22 (*n* = 12 boys; *n* = 10 girls) Ethnic groups: Italy, Morocco, Brazil, Nigeria, Bolivia, India, China, Spain
	Educational team	19 professionals of the educational team:	^*^Teacher tutor: *n* = 10; Specialist teacher: *n* = 5; Support teacher: *n* = 4
Interview focus group	Management team	25 education professionals:	Director: *n* = 2; Head of studies: *n* = 2; Consultant: *n* = 2 ^*^
	Educational team	6 professionals del management team:	
		19 professionals of the educational team	

^a^Within the students, there is a distinction between 3rd (*n* = 75) and 4th grade (*n* = 69) students. Among the 25 education professionals, two groups are distinguished: management team (*n* = 6) and educational team (*n* = 19). The management team consists of the figures of the director, the head of studies and the advisor. Educational team consists of 19 professionals with different profiles such as teacher tutor, specialist teacher (English teacher, physical education teacher, Spanish language teacher and music teacher) and support teacher (therapeutic pedagogy teacher, support specialist and consultant).

Ninety observation sessions (30 sessions in each school center during 3 months), 17 individual interviews and 2 focus groups were conducted. Observation sessions were carried out in the classroom, dining room and recess areas.

In order to facilitate the observation process, we began with the categories proposed by LeCompte and Goetz (1993), which are those frequently used in ethnographic research (i.e., who, what, where, when, how and why).

With regard to the interviews and focus groups, considering the diversity of the professional and personal profiles of the participants and the different working conditions in which they found themselves, the type of interview used in this research was semi-structured and interviewer-administered. Following Flick's indications (Flick, 2014, 2018), both the interviews and focus groups were flexibly designed and developed, formulating the questions and controlling the pace of the interview and the focus groups according to the responses of the interviewees, frequently altering the order and form of the questions and adding new questions if necessary. The main topics addressed in the interviews were the following: cultural diversity, specific educational support needs, information on basic developmental needs and information on care, the state of the teaching staff, the school's relations with the administration, the teaching-learning process, coexistence, types of communication, academic performance and evaluation, and the use and management of time and space.

Interviews and focus groups were audio-recorded with prior consent. The Ethics Committee in Human Research at the University of Basque Country approved this study with the following identification: M10_2019_134.

### 3.3 Data analysis

After the transcription of all the observation sessions collected through the field notes, interviews and focus groups, the information was analyzed. To ensure the analysis with greater accuracy, the information was coded using NVIVO-12 software, in which the participant, the school and the source of information were identified (see Table 2). The coding of the information was carried out in several phases. First, open coding was applied, which allowed for the identification of relevant units of meaning in the participants' discourses and observations. Subsequently, axial coding was carried out, grouping the initial codes into broader categories that reflect recurring patterns regarding students' emotional stability, the use of authority, inclusive practices and other resources, and dynamics of school segregation.

**Table 2:** Information coding.

**Instrument**	**When**	**Where**	**Who**
Diary	Diary: D (classroom observations)	CA: Center A CB: Center B CC: Center C P: Patio C: Canteen	S: Student Coor: Coordinator T: Teacher tutor SPT: Specialist teacher ST: Support teacher Dr: Director HS: Head of studies Ct: Consultant
Interviews	Interview number: I1-In		
Focus groups	Group: FG1-FGn		

In order to ensure the credibility and reliability of the qualitative analysis, rigorous triangulation procedures were implemented both methodologically and among researchers. Methodological triangulation was carried out by contrasting data obtained from various sources —field notes, interviews, and focus groups— which enriched the interpretation and strengthened the internal validity of the study (Denzin and Lincoln, 2017; Flick, 2014). At the same time, triangulation among researchers was conducted through cross-reviewing the coding by the three team members. Each researcher coded and analyzed the information in an inductive-deductive manner, and subsequently, the codes, categories, and selected information were compared and the most relevant areas were integrated into common categories.

Finally, to ensure the confidentiality and anonymity of all participants, all names used in the Results section are fictitious (Abad, 2016).

## 4 Results

Two emerging categories emerged which reflect the determining factors that hinder the achievement of a truly inclusive school (see Table 3). The first three themes are framed within the first category that refers to *Pedagogical relations in the framework of inclusive schooling*. The remaining two themes fall into the second category, *Teachers‘ discomfort toward different aspects*. These categories, and the themes and sub-themes that define each of them are described below:

**Table 3:** Master list of categories, super-ordinate and sub-themes.

**Categories**	**Super-ordinate themes**	**Sub-themes**
Pedagogical relations in the framework of inclusive schooling	Emotional instability of students	Difficult family situation. Coverage of some basic needs questioned. Overrepresented ethnic minority groups in child protection services Revelation of affective deficiencies in relational models. Externalizing behaviors.
	The crisis of authority	Reminding students to obey the rules. Questioning of the authority. Different positions regarding the notion of authority.
	Good teaching practices	Emotional support. Positive emotional competencies. Difficulties.
Teachers‘ discomfort toward different aspects	Student grouping and segregation	Discrepancies with official data. Ethno-cultural diversity. School segregation and ghettoization. Xenophobic behavior of the families. Families as highly socially vulnerable.
	Teachers need other resources	Impact of labor instability on project implementation. Impact of labor instability on teacher-student relations. Insufficient resources. Need for other support. Lack of coherence between the training received and the realities of their schools.

### 4.1 Theme one: emotional instability of students

Close affective relationships help understand, express, regulate and make appropriate social use of emotions, providing the individual with emotional security (Smith, 2019). By contrast, insecure attachment relationships lead to emotion regulation difficulties, which might be associated with interpersonal difficulties, discomfort with intimacy, and behavior problems (Godbout et al., 2019).

#### 4.1.1 Difficult family situation

All the interviewed teachers agreed that a large number of students, approximately 80% of the participating students, have a difficult family situation and this family situation influences their behavior in class creating stressful situations.

Normally everyone comes with a backpack, with a family backpack. In the end, they spend a third of their day here, so it's noticeable. Yes, it is very noticeable. If the family doesn't have stability at an emotional level, this is reflected in the child. [T, CC, FG2]

#### 4.1.2 Coverage of some basic needs questioned

Certain dynamics and unstable characteristics of the family system of many students made some teachers question the coverage of basic needs related to sleep, care, emotional stability and cognitive stimulation, sometimes even using a critical tone toward families by using words such as “dysfunctional.”

The tutor tells me that, with the exception of four families, the rest are dysfunctional. He explains that Bassim's father is in prison because he broke the restraining order and beat up his wife. Bassim witnessed this scene. “In this class, children normalize it, because everyone is in the same situation,” concludes the teacher. [T, CC, D]

#### 4.1.3 Overrepresented ethnic minority groups in child protection services

In many cases, pupils were detected and diagnosed with specific educational support needs or were being monitored by other institutions such as social services or mental health services (e.g., psychiatry). In fact, ethnic minority groups are overrepresented in child protection services (Arruabarrena et al., 2019). This harsh reality is reflected in some of the interviews conducted.

The consultant sends us an email every 2 months to collect information for social services. We have a high percentage of children in social services in class, and then social services come here every 2 or 3 months. They meet with the consultant and talk about all the cases. [T2, CA, I4]

#### 4.1.4 Revelation of affective deficiencies in relational models

In other cases, despite not having any diagnosis, many children in these schools revealed affective deficiencies in their relational models. A particular case was observed within the student population that required greater attention and dedication by teachers, given that his reactions, which derived from the discomfort he suffered, constantly interrupted the rhythm of the class.

John waits and, in the meantime, starts to bother his classmates by interfering with their view of the digital whiteboard. Several annoyed people shout his name: “John!” and he gets angry and leaves the classroom abruptly, slamming the door and starting to bang on it from outside. [S, CA, D]

#### 4.1.5 Externalizing behaviors

Generally, the students' discomfort is manifested by impulsive, aggressive or defiant behaviors, which usually generate discomfort or disturbance in his/her environment.

Keidan does not tolerate limits and very furious (frown and mouth furrowed and with a threatening look), he throws the papers at Fede, the tutor, and says: “You bastard!” [S, CC, D]

### 4.2 Theme two: the crisis of authority

Whatever the cultural nature of people, any group of people is always diverse, so the implementation of democratic rules, characterized by high levels of affection and control, that facilitate harmony in coexistence is essential (Grau et al., 2016). For this reason, in terms of emotional stability, rules play a restraining role.

#### 4.2.1 Reminding students to obey the rules

In the school context, teachers constantly reminded them which were the social rules through their messages, that is, by telling them how they should behave or how they should act in different spaces, such as in the classroom and in the playground. Thus, in case of non-compliance, teachers were usually strict.

The teacher, in an imperative way, reminds them some rules, some prohibitions: they cannot take out the Sciences book until they hand in the exam; they cannot get up, they have to raise their hands. [T, CA, D]

#### 4.2.2 Questioning of the authority

However, at some difficult moments, authority was challenged. For example, when the previously set rule or limit fell or failed to work and attempts by professionals to re-establish it were sometimes frustrated and their authority questioned.

Amy, the English teacher, tells them without raising her voice: “I'm going to get angry! But it has no effect and they continue with the same defiant attitude. [S, CA, D]

#### 4.2.3 Different positions regarding the notion of authority

The rules are framed within general rules, which are agreed and established in our society in general, and in the particular community to which the students belong. Although nobody questioned the need for coexistence rules, there were diverse opinions regarding their rigidity and forcefulness.

Throughout the interviews and focus groups, teachers showed different positions regarding the notion of authority and openly shared the questioning of it. This showed the lack of consensus in the educational community.

I think that the crux of the matter is the balance between confidence and authority, which in my opinion is the biggest challenge we teachers face. This balance has to be maintained, because when you are too confident, children don't respect you and when you are too authoritarian, they are afraid of you. And not all teachers manage this balance. [Dr, CA, FG1]

Authority now is very devalued. How many times have we commented among ourselves that the child is not respecting the limits and that the grandfather, grandmother, father or mother… doesn't say or do anything? I think authority is misunderstood. [S2, CA, FG1]

It has happened to most of us that, having had parents with a strict upbringing, we now have children with whom we have somehow not been able to assume authority. [Dr, CA, FG1]

### 4.3 Theme three: good teaching practices

The characteristics of these schools, which were largely produced and determined by school segregation (Reardon and Owens, 2014), generated a myriad of problematic situations in the area of daily coexistence that made it difficult for teachers to intervene. However, despite these shortcomings and deficiencies in the system, thanks to the commitment and tenacity of teachers, they always ended up responding to the needs and demands of their students.

#### 4.3.1 Emotional support

The emotional support provided mainly by the tutor used to be a source of calm for pupils. As the following voices show, this emotional support was usually carried out with a calm and close attitude. Generally, after the emotional support provided by the reference person, children felt more emotionally stable.

Adel gets frustrated and cries, he can't draw the Christmas tree he is drawing. The teacher restrains him and provides him guidelines on how to draw it correctly. Adel restrains himself and continues to work calmly. [S, CC, D]

#### 4.3.2 Positive emotional competencies

It was essential for teachers to have positive emotional competences (e.g., adequate emotion regulation strategies), in order to emotionally contain pupils in the teaching-learning process and in the process of emotional maturation.

Monique is not calm; she can't stay still in her place. She approaches the blackboard more than once. She cleans her desk. She approaches the blackboard again, and although she makes mistakes, with the help of Ann, the Spanish teacher, she ends up answering correctly. [SPT, CB, D]

#### 4.3.3 Difficulties

Considering students' emotional needs, implementing strategies to help them balance their emotional needs was another way of providing care. Nonetheless, in the more complex cases, despite the good intentions of teachers, it was not easy to access the inner world of the students.

At the beginning of the course the class was quite horrible, everything was characterized by disobedience and defiance. Then it has relaxed quite a lot, especially because we have made a thorough follow up with families. But it is very frustrating. [T2, CA, I4]

### 4.4 Theme four: student grouping and segregation

Based on the diversity that made up these more vulnerable social realities, in any of these schools there was a high percentage of students from different ethno-cultural backgrounds.

#### 4.4.1 Discrepancies with official data

However, the realities of the schools did not always coincide with the official data.

The difference is that the Basque Government only takes into account pupils who were not born in Spain. For us, a person, a child, immersed in the community but who is not integrated or who is on the fringes of society, and therefore, does not have a relationship, is not integrated. He/she is still a foreigner, even if he/she was born here, because society treats him/her as a foreigner. [HS, CC, I13]

#### 4.4.2 Ethno-cultural diversity

The existent ethno-cultural diversity in schools was one of the aspects most pointed out in the interviews held with the teaching staff. In all the observed classrooms there were children from different ethnic and cultural origins: African, North African, Asian, Latin American, and Romany. It is worth noting that many of these children were born in Vitoria-Gasteiz. However, there were also children of migrants, mostly from African (50%, e.g., Cameroon, Senegal, Morocco, Algeria); Latin American (20%, e.g., Bolivia, Mexico, Colombia, Paraguay); Asian (10%, e.g., China,); and different European countries (10%, e.g., Italy, Ukraine) and from other communities in Spain (e.g., Galicia, Andalusia).

I notice the multi-ethnicity of the children in this class. There are five Africans, nine Latin Americans, three North Africans, one Asian and five Basques. [S, CC, D]

#### 4.4.3 School segregation and ghettoization

The ethnic diversity leading to the presence of a great cultural diversity is the sweet side of a larger problem referred to by the staff of these segregated schools. The phenomenon of segregation, both at school and in society, goes hand in hand with another social phenomenon, known as ghettoization (Felouzis et al., 2018). According to the following testimonies, the administration has not yet managed to respond to this problem.

I think that heterogeneity is always important, not having separation. What is happening here is that there is heterogeneity in the primary school classes, there are children from Vitoria as well as from Nigeria. There is a mixture of everything, which is what enriches a group. But we are realizing that for example, in the two- and three-year-old classes, the majority of children are from other cultures. This makes the school very difficult and also stigmatizes it. In fact, some families from here don't enroll their youngest child here and send him or her to another school. [Dr, CA, I1]

This school catches your attention. In the end, this school is very ghettoized. The problem is not only with this school, but also with the Basque education system, that is, with the way they treat public schools. [HS, CC, FG2]

#### 4.4.4 Xenophobic behavior of the families

Thus, on more than one occasion, teachers highlighted the differences between different schools, public and charter, in terms of the ethno-cultural diversity of their pupils and families. For some people, the root causes had to do with the xenophobic behavior of the families that used to belong to these schools.

When the locals saw that foreigners were coming, they left and that's why segregation has increased. Most of the people who have come here are from the Maghreb, mostly from Morocco, less from Algeria and then from West Africa, Nigeria, Mali and also Guinea, Senegal and Burkina Faso. Above all, from these areas. We also have Chinese, but less, Latinos too, but not many. [HS, CB, I7]

#### 4.4.5 Families as highly socially vulnerable

These are the reasons why the three schools coincided on describing their students' families as highly socially vulnerable, mainly as a result of economic precariousness and lack of resources:

I think that what affects us most is the economic aspect. Because there are families, for example, Nigerian families, who are very polite. And there are others that create disruptions. But I think it's more about the economic and cultural context than about the origin. [ST2, CA, FG1]

### 4.5 Theme five: teachers need other resources

Complaints from teachers about the education administration were constant. Education professionals denounced the lack of both human (e.g., job instability and shortage of specialized staff) and material resources (e.g., lack of adequate in-service training).

Job instability and the consequent mobility of teaching staff is a consequence of different factors, such as sick leave or retirements. However, it is mainly due to the type of contract, which is generally temporary, and the consequent instability in the jobs.

#### 4.5.1 Impact of labor instability on project implementation

Apart from other negative consequences, this instability also hindered the implementation of cross-cutting projects designed to promote a more respectful and inclusive coexistence, or the implementation of new methodologies that would imply a change in the ordinary model of praxis.

We started working on cooperative learning, but of course, because there is so much change in the teaching staff, even if you do the training, it's not possible to implement it in the classroom because people come and go. [HS, CA, I2]

#### 4.5.2 Impact of labor instability on teacher-student relations

In one of the schools, during the time in which the observations were carried out, the figure of the educational support specialist for one of the children with special educational needs was changed on three occasions. Therefore, the job instability promoted by the current central administration not only hindered the implementation of innovative projects and the management of the school, but it also had a direct influence on the relationship with the pupils, with the consequent repercussions at both an emotional and cognitive level.

Of course, it has a negative impact. They are children and they get used to a teacher. That's good because that person takes care of them, knows them and knows how to handle them. On the other hand, if every year a new person comes, who has to learn from scratch, it's like a lost job. Families also have a hard time. [T2, CA, I4]

#### 4.5.3 Insufficient resources

Similarly, teachers were generally dissatisfied with the resources currently available. Despite the fact that pupils had previously been identified as having Specific Educational Support Needs due to the difficulties they showed in their maturational development, the resources allocated by the administration were not sufficient to attend all of them. As a result, some of their specific needs remained unmet or the educational team had to make real sacrifices in order to try to ensure that the quality of the teaching and learning processes were affected as little as possible.

The allocation of resources by the Department of Education is made according to a forecast and it is allocated for the following year (Basque Government, 2020). However, as the following voice explains, it may happen that enrolment keeps moving and that in that period of time a child with a diagnosis of special educational needs comes to the school. In this case, the resources allocated to these pupils remain in the previous school they leave.

You get the child, but not the resource. You have to manage with the 4 that they give you. So, there are times when… it seems to me that they are not attending us in the way they should. [Dr, CA, I1]

#### 4.5.4 Need for other support

Due to the complexity of the problems of these schools, several interviewed people directly demanded other support systems, such as the services of a psychologist.

There is a terrible suffering. I mean, they have a very big backpack. A child doesn't do the things he/she does just because. There is something there that we have to keep digging to see where it comes from. And we do notice that we would need, for example, the figure of a psychologist, someone to accompany the child. Because here, even if we try, we don't have the resources to do so. [Dr, CA, I1]

#### 4.5.6 Lack of coherence between the training received and the realities of their schools

Teachers also expressed their unease about the lack of coherence between the training offered by the administration and the realities of their schools. As a result, given the dissatisfaction with the training received and the real needs of these schools, teachers often voluntarily undertake complementary training outside the compulsory training they are required to undertake by the education administration.

The administration doesn't give us the kind of training that is useful to us at an educational project level, because it doesn't know our project. The administration has offered its courses for a long time and these are not always adjusted to our reality. So, the training that we prepare is perhaps more valid for ourselves. We work with pedagogical, dialogical gatherings, with a text or a book as a basis, which allows us to talk about the situation we have in the classroom. [HS, CC, FG2]

## 5 Discussion and conclusions

This study has analyzed the protective and vulnerability factors that condition the teaching-learning process and the socio-emotional development of children at risk who attend primary schools. Despite the resilience shown by some of these children, the results show that the diverse symptoms presented by most of them hinder interactions between people, which often triggers stressful environments for coexistence that impedes the teaching-learning processes. Likewise, social segregation and job instability characteristic of these schools, high student-to-class ratios, and a lack of specialized staff are some of the factors identified in this study that might be an obstacle for the achievement of a more inclusive school.

The results of this investigation provide valuable information on the consequences of ACE on children's wellbeing. As the results have shown, the behavioral and physical manifestations of toxic stress vary from child to child. Therefore, detecting cumulative ACE exposure is a fundamental first step in identifying children at risk of developing a negative response to adverse experiences (Oh et al., 2018). In this sense, early detection of problems associated with vulnerability could be key to implementing appropriate interventions to prevent relationship dynamics that could expose children to dangerous and abusive situations in the future.

The findings also show the impact of school segregation on both children's socio-emotional development and their learning process. However, despite the existence of a vast scientific literature (Buchanan-Pascall et al., 2018; Mundy et al., 2017; Reardon and Owens, 2014) that highlights the risks of bringing together students from disadvantaged socioeconomic backgrounds, many education systems continue to intentionally foster the concentration of students with similar social characteristics in schools and classrooms (Nusche, 2009; Pàmies and Tarabini-Castellani, 2017). This is the reality of the three schools analyzed, and, as has been shown, the social factors that permeate the structure of these schools have a negative impact on children's academic performance and school failure. This is probably due to two main reasons: firstly, they underachieve compared to other non-at-risk children because they generally have access to fewer socioeconomic resources; and second, these individuals are disadvantaged by suffering a penalty associated with their social class (Rothon, 2007).

In order to ensure their wellbeing, teachers indicated that they constantly promoted optimal pedagogical relationships through empathy, assertiveness, authority and listening, to mitigate the effects of the vulnerability in which some of the children attending these schools find themselves. In this vein, our research findings also show that teachers are mainly emotional managers.

The data obtained in this research also shows that the educational context is a clear reflection of the current global crisis. Now, in times of globalization and as a consequence of neoliberal policies, new relational models are being established that question, among other issues, authority in all its spheres (Narodowski, 2012; Recalcati, 2016). The interviewees in our study advocated a horizontal and non-hierarchical type of pedagogical relationship. Thus, there is an increasing trend toward a democratic style of authority with a pedagogical purpose (Brandoni, 2017; Freire, 2002) in which rules are reasoned and agreed with the students. Nevertheless, especially at times characterized by chaos and disorder, rules are widely used in a directive way, without questioning the pedagogical purposes of rules. In fact, according to the testimonies collected through the interviews and focus groups, it seems that most people confuse the notion of authority with authoritarianism.

This lack of definition with regard to the notion of authority generates confusion. With all this, one idea that came up repeatedly, explicitly or implicitly, was that the concept of authority is in crisis, not only in the educational community but also in society in general. Going back to past times of censorship and repression^1^, the general feeling is that we have moved from oppressive authoritarianism to total freedom. Through a more permissive educational style (i.e., high levels of affection and low levels of behavior control), there is no clarification of the places, roles and functions that define people according to their stage of development. Likewise, in agreement with other studies (Blodgett and Lanigan, 2018; Briggs and Hawkins, 2020), our findings suggest that education professionals are not always able to meet the demand for attention and the search for approval and affection from children who present socio-emotional instability associated with special developmental needs. In our study, this was often reflected as a source of frustration, stress and insecurity for teachers, what made it clear that the teachers' involvement went beyond the merely work-related aspects.

As a consequence, from a systemic perspective, beyond the individual factors affecting these children, other factors must be considered for a global analysis of their situation, such as the lack of material resources of schools; lack or job instability and mobility of teachers; and lack or poor academic training of families (Pérez and Cely, 2004; Rowsell et al., 2017). Given the psychosocial difficulties that these children go through, many of them present emotional instability. In addition to this, the continuous rotation of professionals perceived in the analyzed schools could be considered a bad institutional practice that increases the damage and the consequent initial suffering of these children.

## 6 Implications for theory, research, and practice

This research contributes to the necessary knowledge about the protective and vulnerability factors that influence the teaching-learning process and the socio-emotional development of at-risk children. Thus, it highlights a comprehensive approach of inclusive education that transcends the school environment and extends to the entire society, including public administrations, in order to ensure social wellbeing and respect for interrelated rights. Specifically, the findings of this study show that in a context of constant change, educational policies and teaching practices should be oriented toward inclusion, breaking the links between disadvantage and academic failure. Furthermore, to advance in this direction, the results highlight the essential need to strengthen teacher training in socio-emotional aspects, foster dialogue between students and teachers, and provide psychosocial support, recognizing children and young people as active participants in the construction of a truly inclusive education. Therefore, these recommendations aim to combine educational support with mental health support so that educational practices are trauma-informed, utilizing socio-emotional learning techniques, and foster a safe, supportive environment.

The findings of our study highlight the need for greater political involvement. Specifically, it is necessary to promote public policies that encourage collaboration between schools and psychosocial and mental health services. The response to the right to inclusive education is not only the responsibility of the school, but of society as a whole, that is, from public administrations to society as a whole (Ainscow et al., 2013; Fedulova et al., 2019). A more holistic approach is therefore needed in order to enable the idea that rights related to social welfare are interrelated and interdependent, since for rights to be autonomous, these must depend on the system that restrains them. Public policies should be oriented toward an approach where the reciprocity between the different rights in the same interrelated process matters (Lara, 2017). For example, intervention programs should be funded and legislative frameworks should be created in order to facilitate inter-institutional coordination, so that children and families receive this support by both, schools and social services.

In addition, given the rapid changes that take place in our society, in order to cope with these changes, it is necessary to shift toward inclusive education that takes into account various psychosocial aspects, such as the individual characteristics of children and the characteristics of their environment (Khambati et al., 2018; Larsen and Holsen, 2021). Thus, a major challenge for policy leaders and practitioners is to find ways to end the links between disadvantage, school failure and conditioned life chances (Ainscow, 2010). This is in line with one of the specific sustainable development goals of the 2030 Agenda, which is to achieve inclusive, equitable, and quality education (United Nations, 2025).

## 7 Limitations and future recommendations

In spite of this study's contributions it is limited in several ways. One limitation has to do with the outbreak of the pandemic. This study started before the pandemic and has now been completed. The confinement of the participants probably affected their emotional and behavioral responses. The effects of the measures taken by the pandemic have not been analyzed. In addition, after the release from confinement and return to school there was one school that refused to participate in the focus groups. They reported being overwhelmed by the new measures put in place by the administration. Furthermore, this research has not measured the possible impact of school segregation. Therefore, a future extension of this research should take into account the different factors that characterize school segregation (e.g., income level) in both data collection and analysis. In this regard, it would be particularly interesting to analyze the impact of school segregation on the cognitive and socio-emotional development of at-risk children. Another limitation refers to the linguistic diversity of the participating population, since the impact that different linguistic realities may have had on communication and interactions between individuals has not been analyzed.

Likewise, there is a need for further research in resilience mechanisms, along with protective factors and interventions in stressful contexts such as segregated schools. In this vein, one possible line of research could be to delve more deeply into the principle of teacher authority, trying to find out the difficulty of putting into practice those aspects that the teachers themselves demand. These refer to a democratic and legitimized authority, along with an inductive discipline at school that provides social support and secure emotional bonds as protective factors.

In order to make further progress in favoring the inclusive school, one line of research and intervention could be based on investigating further the good teaching practices that are already being carried out in these schools. Despite the structural difficulties they have to face on a daily basis, the entire educational community of these schools makes an effort that is sometimes extraordinary and generally insufficient to make inclusive schooling a reality. To this end, we recommend specific training that is more focused on the needs related to children's socio-emotional development, which is essential to carry out good teaching practices and make early detection possible in those cases that require it. In line with Messiou (2019), a cultural shift in education that recognizes children and young people as central agents in shaping pedagogical thinking, policies, and educational practices is proposed. Specifically, this author emphasizes the importance of dialogue between students and teachers as a means to co-construct meaningful teaching and learning processes. For the proper functioning of teaching and learning processes, we also consider it necessary to offer teachers supervision and psychosocial support.

In summary, our results highlight that education and schools are faced with the challenge of achieving equity, so that all students have the same equal and quality of opportunities for their all-round development (Qvortrup and Qvortrup, 2018). However, true inclusion of all people will not be achieved if countries do not fulfill their commitment to make their schools more inclusive (Ainscow et al., 2013).

## Data Availability

The raw data supporting the conclusions of this article will be made available by the authors, without undue reservation.

## Footnotes

1 The authors refer to the Dictatorship of Francisco Franco or Francoist Spain, the period from the end of the Spanish Civil War in 1939 until his death in 1975.
